# Supplementary material for: Blood transcriptome analysis suggests an indirect molecular association of early life adversities and adult social anxiety disorder by immune-related signal transduction
Source: Front Psychiatry. 2023 Apr 25;14:1125553. doi: 10.3389/fpsyt.2023.1125553 (PMC10168183; doi:10.3389/fpsyt.2023.1125553)
Supplement: Supplementary file 1 [file Data_Sheet_1.docx]

Supplemental figures


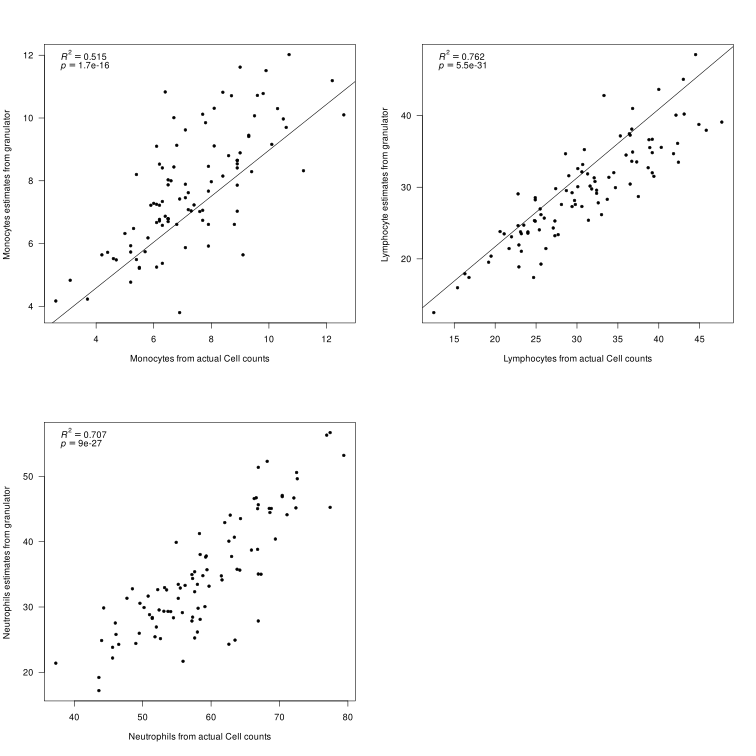
**Figure S1**: Correlations of cell type ratios estimated using *granulator* and real counts from a cohort subset for **A** Monocytes and **B** Lymphocytes.

A

B

*
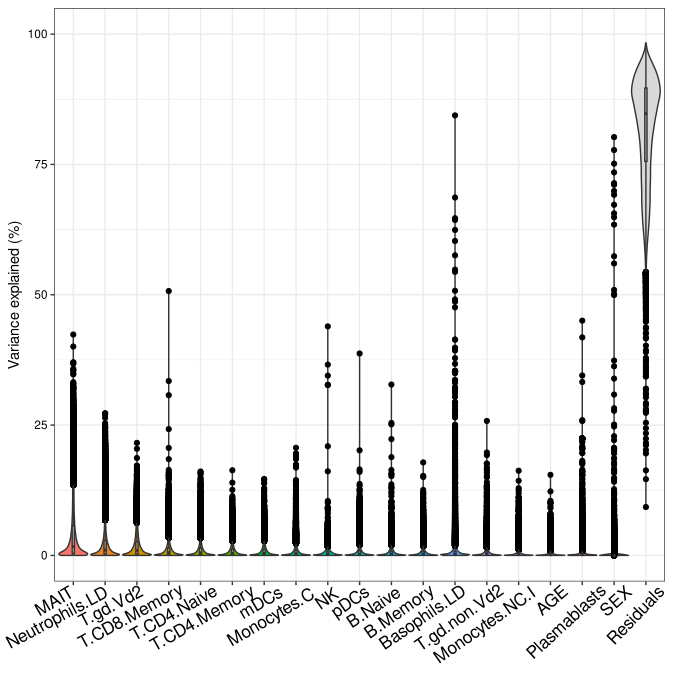
*

**Figure S2**: Explained variance by variables in normalized gene expression prior to gene count adjustment.


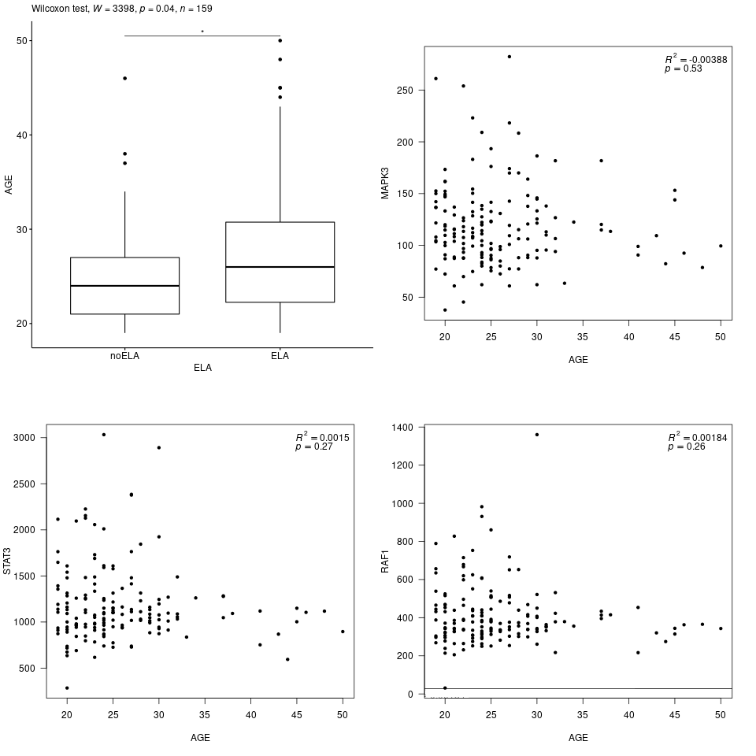


A

C

D

B

**Figure S3**: **A** The age distribution is shown among the groups with high and low levels of ELA, respectively. However, there is no correlation of age and gene expression of representative genes, namely **B** *MAPK3*, **C** *STAT3* and **D** *RAF1*.


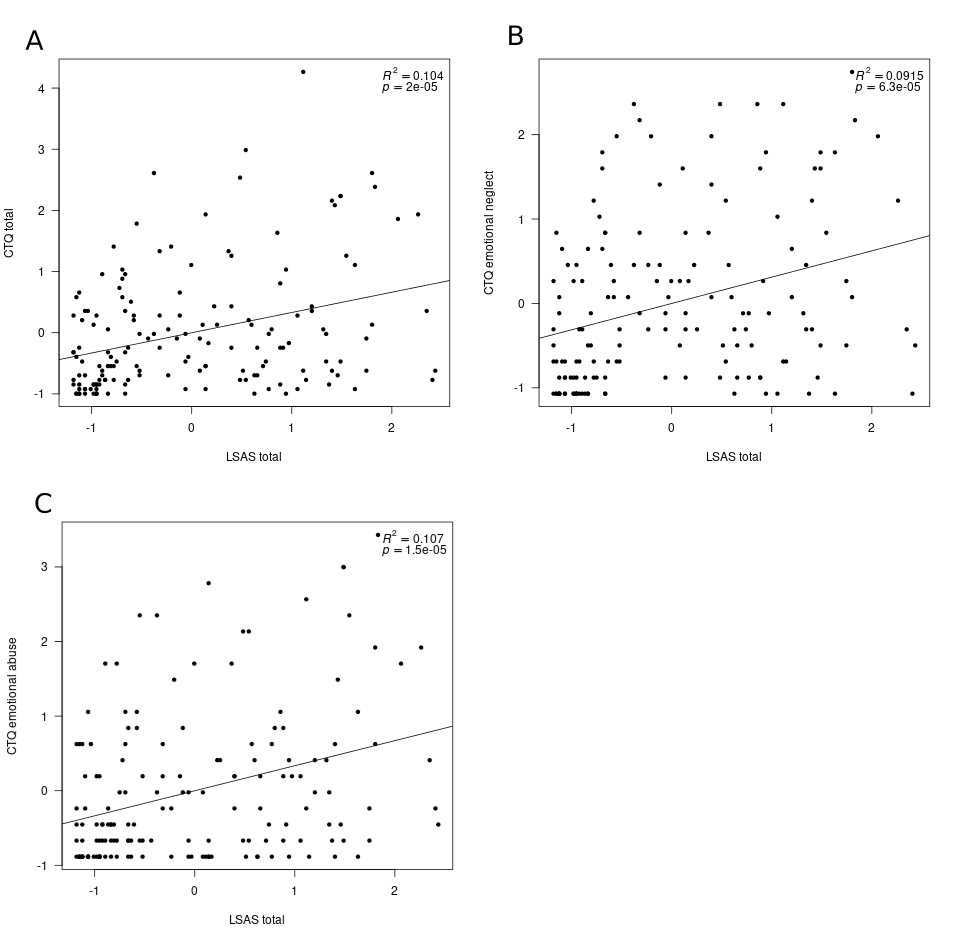


**Figure** **S4**: Correlations of scaled CTQ and LSAS score with **A** CTQ total, **B** emotional neglect and **C** emotional abuse.


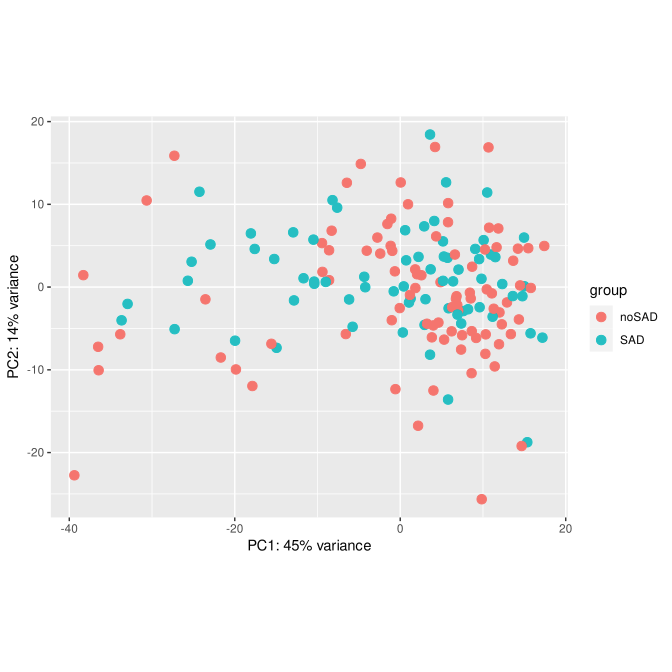


**Figure** **S5**: Principal component analysis of normalized and cell type ratio adjusted gene expression data with respect to SAD.


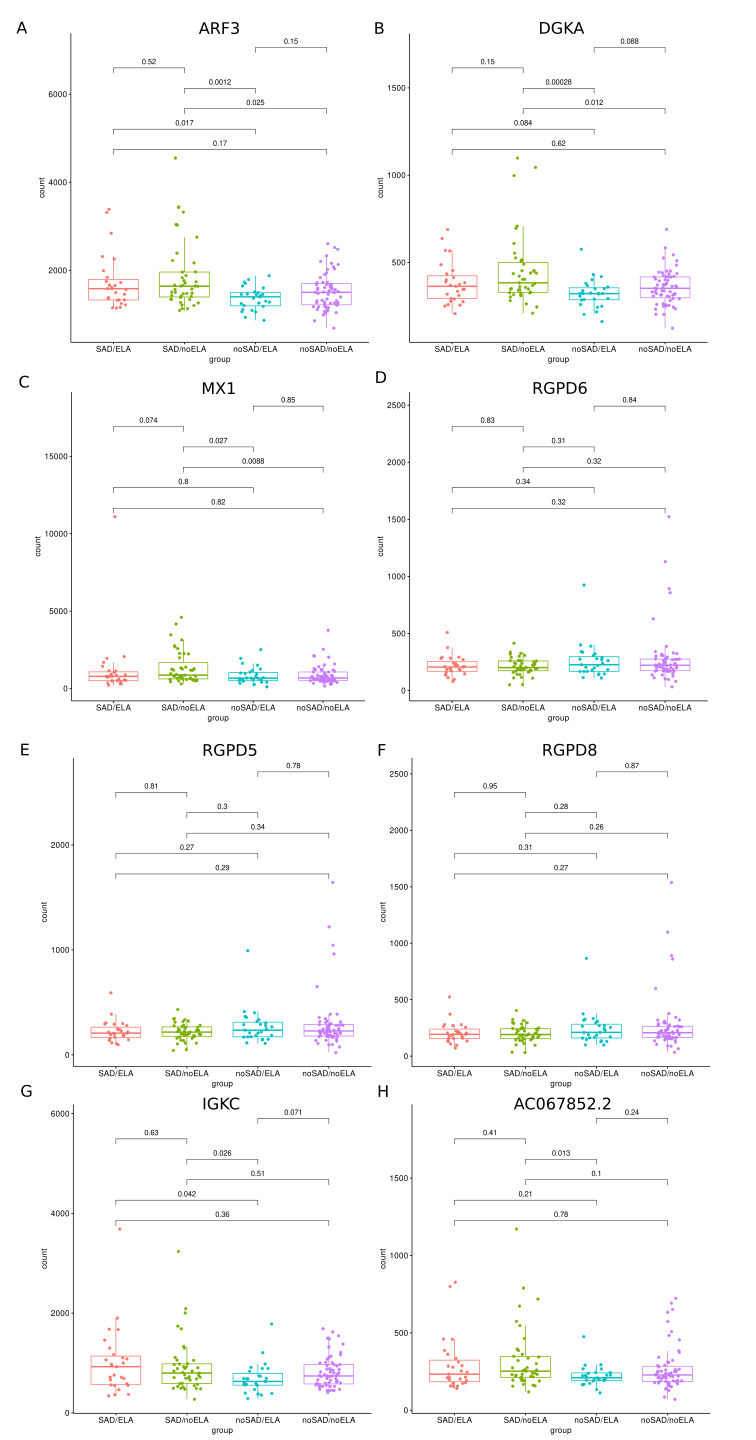


**Figure** **S6**: Gene expression patterns of the eight significantly differentially expressed genes (DEGs) between the SAD group and controls that exhibited an expression rather caused by outliers or other effects than being a true DEG. The normalized and cell type adjusted counts are shown with respect to SAD and ELA. The Wilcoxon rank sum test was applied and the p-values were adjusted for multiple testing using Benjamini-Hochberg correction. Expression patterns are shown for **A** ADP-ribosylation factor 3 (*ARF3*), **B** Diacylglycerol Kinase Alpha (*DGKA*), **C** MX Dynamin Like GTPase 1 (*MX1*), **D** RANBP2 Like And GRIP Domain Containing 6 (*RGPD6*), **E** RANBP2 Like And GRIP Domain Containing 5 (*RGPD5*), **F** RANBP2 Like And GRIP Domain Containing 8 (*RGPD8*), **G** Immunoglobulin Kappa Constant (*IGKC*) and **H** the long non-coding RNA *AC067852.2.*


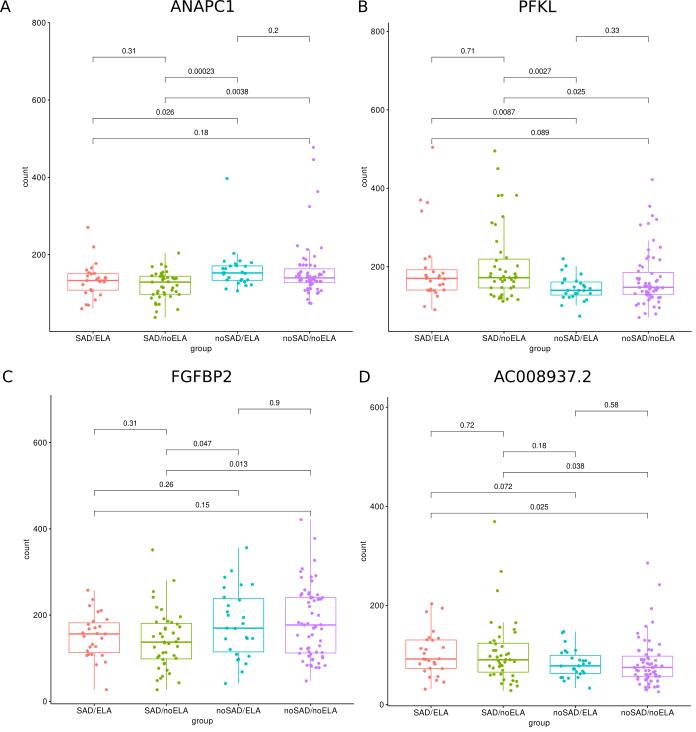


**Figure S7**: Gene expression patterns of four significantly differentially expressed genes (DEGs) between the SAD group and controls showing a clear SAD associated expression pattern. The counts are shown with respect to SAD and ELA. The Wilcoxon rank sum test was applied and the p values were adjusted for multiple testing using Benjamini-Hochberg correction. Expression patterns are shown for **A** Anaphase Promoting Complex Subunit 1 (*ANAPC1*), **B** Phosphofructokinase, Liver type (*PFKL*), **C** Fibroblast Growth Factor Binding Protein 2 (*FGFBP2*) and **D** the long non-coding RNA *AC008937.2*.


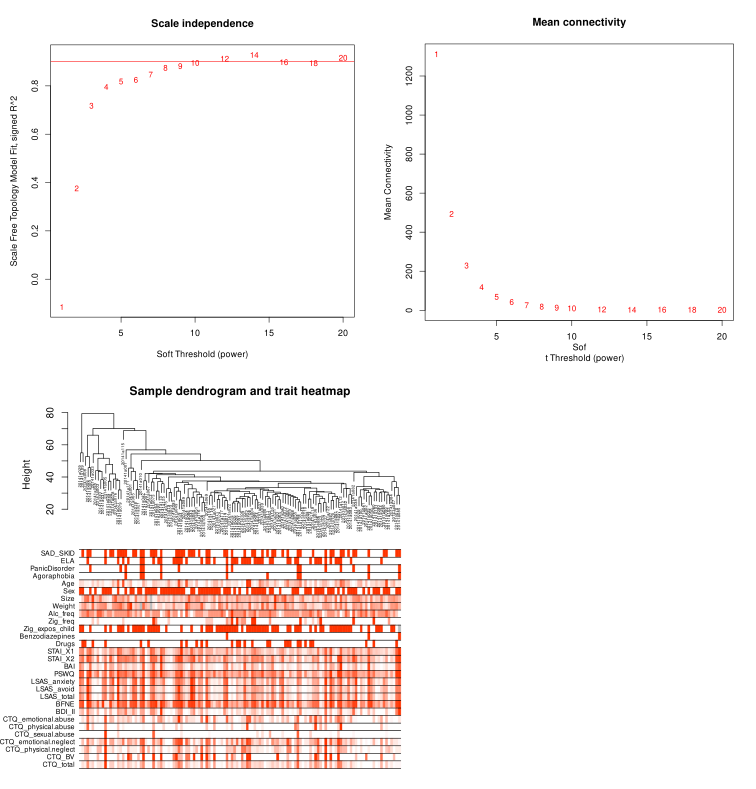


**Figure S8**: Weighted Gene Co-expression Analysis (*WGCNA*). **A** Selection of the soft threshold by Scale-free topology fitting index R2 analysis and **B** mean connectivity for various soft threshold powers. **C** Sample dendrogram and trait heatmap revealed no severely deviating samples.


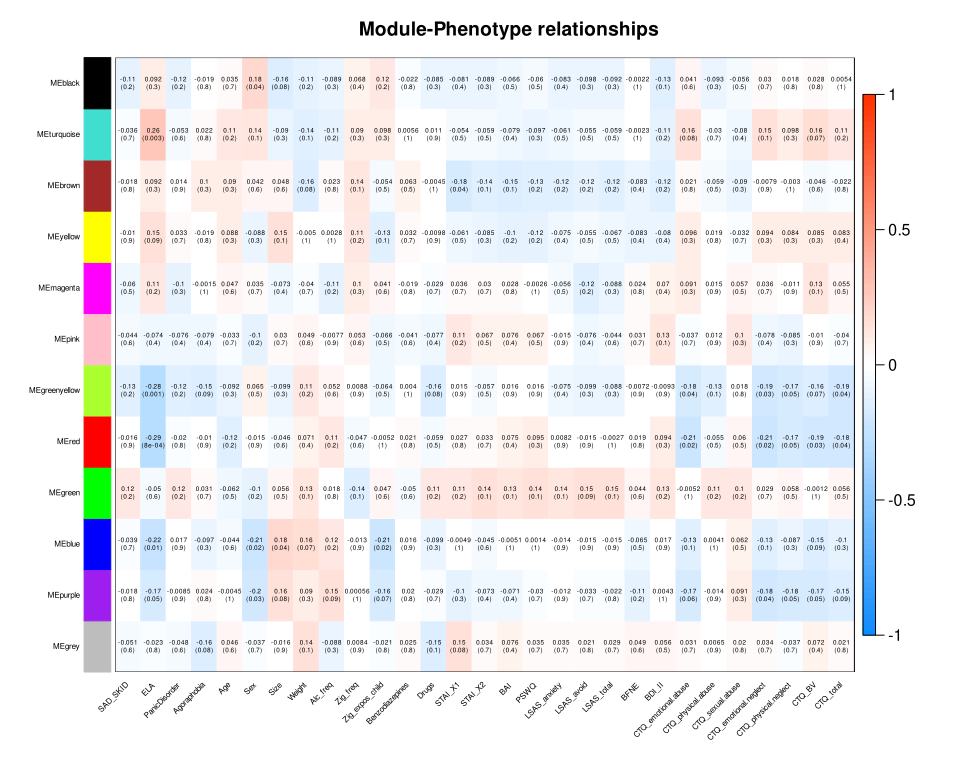


**Figure S9**: Heatmap of the correlation between the variables and module eigengenes. Correlation coefficients are followed by p-value in brackets.

**Figure S10:** Gene functional enrichment of genes overlapping between the MAPK3 ppi String network and the turquoise *WGCNA* co-expression module.
